# Translation and Validation of the Persian Version of the Communication and Language Assessment Questionnaire for Persons with Multiple Sclerosis (P-CLAMS)

**DOI:** 10.1093/arclin/acaf060

**Published:** 2025-06-30

**Authors:** Aghaei Fatemeh, Rahmani Shima, Azarinfar Maryam, HaresAbadi Fatemeh, Ghaemi Hamide, El-Wahsh Sarah

**Affiliations:** Contemplative Studies Centre, Melbourne School of Psychological Sciences, Faculty of Medicine, Dentistry, and Health Sciences, The University of Melbourne, Melbourne, Victoria, Australia; Department of Speech Therapy, Paramedical Sciences Faculty, Mashhad University of Medical Sciences, Mashhad, Iran; Department of Speech Therapy, School of Rehabilitation Sciences, Isfahan University of Medical Sciences, Isfahan, Iran; Department of Speech Therapy, Paramedical Sciences Faculty, Mashhad University of Medical Sciences, Mashhad, Iran; Department of Speech Therapy, Paramedical Sciences Faculty, Mashhad University of Medical Sciences, Mashhad, Iran; Department of Communicative Sciences and Disorders, Michigan State University, East Lansing, Michigan, USA; Discipline of Speech Pathology, Faculty of Medicine and Health, The University of Sydney, Camperdown, NSW, Australia

**Keywords:** Communication disorders, Patient-reported outcome measures (P-CLAMS), Multiple sclerosis, Questionnaires, Speech-language pathology, Persian language

## Abstract

**Objective:**

Persons with multiple sclerosis (MS) can experience communication changes, which can significantly impact their quality of life. To explore and help address these challenges, patient-reported outcome measures (P-CLAMS) can be valuable tools in both research and clinical practice. They can help track symptom progression, support patient-centered care, assess the effectiveness of service delivery, and complement clinical assessments. This study aimed to translate and validate the Persian version of the Communication and Language Assessment Questionnaire for Persons with Multiple Sclerosis (P-CLAMS).

**Method:**

The adaptation and translation of the questionnaire occurred in two phases: (1) translation and cultural adaptation and (2) validity and reliability. The final version of the P-CLAMS consists of 11 items with one component factor (communication/language).

**Results:**

Significant differences were observed in construct and criterion validity measures. Confirmatory factor analysis indicated that the P-CLAMS is a unidimensional measure for assessing communication difficulties in MS. The internal consistency was high (α = 0.92), and test/retest reliability was acceptable (ICC = 0.89). Additionally, the P-CLAMS effectively discriminated between people with MS (PwMS) and healthy controls.

**Conclusion:**

The psychometric evaluation demonstrated that the Persian version of the CLAMS (P-CLAMS) has good validity and reliability. The P-CLAMS can be a valuable tool for clinicians and researchers to assess communication changes in PwMS and evaluate intervention effectiveness.

## INTRODUCTION

Multiple sclerosis (MS) is one of the most common progressive neurological diseases worldwide (El-Wahsh et al., 2020a). The clinical manifestations of this autoimmune disease vary according to the severity of nervous system involvement (Harel et al., 2017; Moccia et al., 2017). Despite over 130 years of research since the first diagnosis, the evaluation and treatment of various aspects of MS remain underexplored (Naci et al., 2017; Pierson et al., 2018; Yorkston et al., 2014). While recent advancements have improved our understanding of the disease’s pathophysiology, challenges in comprehensive care and accurate assessments persist (Moccia et al., 2017; Plotas et al., 2023). According to recent studies, approximately 1.3 million people worldwide are affected by MS, with Iran accounting for around 40,000 cases—one of the highest national rates (Eskandarieh et al., 2021; Etemadifar et al., 2024; Moghadam et al., 2021; Sahraian et al., 2019). This growing prevalence highlights the urgent need for specialized diagnostic and therapeutic interventions (Eskandarieh et al., 2021; Mattioli et al., 2020). Evidence also indicates that women are more susceptible to this condition than men (Sahraian et al., 2019).

Communication and cognitive changes in people with MS (PwMS) typically develop gradually and can manifest at various stages of the disease, sometimes even preceding physical symptoms. These changes can include language, speech, and voice alterations, including word-finding difficulty, discourse impairments, difficulty interpreting metaphors and idioms, reduced syntactic complexity, decreased speech rate, unclear vocal quality, and impaired information transmission (Baker et al., 2021; Chiaravalloti & DeLuca, 2008; El-Wahsh et al., 2020b, 2020c; Etemadifar et al., 2024; Hartelius et al., 2023).

Communication impairments in PwMS often manifest themselves in subtle ways and can occur at different stages of the disease. These impairments can include difficulties with word recall, interpreting figurative language, organizing conversations, and maintaining the flow of conversation. They can also include reduced fluency, changes in voice quality, and slower processing speed (Cartwright et al., 2022; El-Wahsh et al., 2020b; Murdoch & Theodoros, 2000; Renauld et al., 2016; Plotas et al., 2023). These deficits typically impact functional communication, interpersonal relationships, emotional well-being, and independence in daily life, significantly affecting quality of life and social participation (Hartelius et al., 2023; Palmer et al., 2016; Yorkston et al., 2014).

Recently, communication changes, prevalent in 60% of PwMS, have gained prominence (Cartwright et al., 2022; El-Wahsh et al., 2020a; Hartelius et al., 2023). Screening and assessment of MS communication skills have been considered for several years but remain ambiguous in clinical settings, possibly because of the need for more accurate and comprehensive tools (El-Wahsh et al., 2020c; Renauld et al., 2016). Patient-reported outcome measures (P-CLAMS) are valuable tools in screening and evaluating intervention outcomes and can provide specialists with access to real-time information for precise decision-making (Chiaravalloti & DeLuca, 2008; Kristjansson et al., 2003).

In 2020, El-Wahsh and colleagues developed a patient-reported outcome measure (P-CLAMS) to assess the cognitive aspects of communication skills in PwMS, called the “Communication and Language Assessment Questionnaire for Persons with Multiple Sclerosis” (CLAMS). This questionnaire is the only comprehensive self-assessment tool available for evaluating communication problems in PwMS, encompassing cognitive, pragmatic, and conversational components to provide a holistic view of communication difficulties (El-Wahsh et al., 2020b). Several Persian questionnaires have been developed for communication (Baker et al., 2021). However, most focus on the psychological and metacognitive aspects of communication skills rather than the specialized evaluation of cognitive, pragmatic, and conversational aspects that the P-CLAMS offers (El-Wahsh et al., 2020b; Kristjansson et al., 2003). To address this gap, the present study aimed to translate, culturally adapt, and evaluate the psychometric properties of the Persian version of the CLAMS (P-CLAMS). Developing a culturally appropriate Persian version of this tool seeks to support clinicians in Persian-speaking regions in enhancing patient-centered care, tracking symptom progression, and facilitating more personalized and effective interventions (Kristjansson et al., 2003). The following study outlines the process undertaken to adapt and validate the P-CLAMS for this population.

## METHODOLOGY

### Ethics

This research protocol received approval from the Ethics Committee of Mashhad University of Medical Sciences under the Code of Ethics IR.MUMS.REC.1400.004. This is a cross-sectional study. Before this psychometric study, written consent agreements were obtained from all patients and healthy participants. The Persian version of the CLAMS was evaluated for its validity and reliability through a two-phase process. The first phase involved translation and cultural adaptation, ensuring the questionnaire was culturally appropriate, and accurately translated into Persian. The second phase focused on assessing the validity of the final translated questionnaire of the P-CLAMS.

### Participants

A total of 95 Persian-speaking PwMS participated in this study between March 2021 and February 2022. Participants were recruited from MS clinics at Qaem and Imam Reza hospitals in Mashhad, Iran. To be included in the study, individuals were required to be 18 years or older and have a confirmed MS diagnosis made by a neurologist specializing in MS based on the McDonald criteria (Thompson et al., 2018). All participants were Persian speakers. The mean age of participants was 35.03 ± 12.02 years, ranging from 18 to 60 years. The study group consisted of 24 men and 70 women. Participants were recruited for this study using a convenience sampling method.

Participants with other neurological disorders, such as epilepsy, stroke, Parkinson’s disease, and traumatic brain injury, as well as those with visual or hearing impairment, were excluded from the study. All participants voluntarily completed an online consent form before completing the P-CLAMS. The questionnaire was distributed via a weblink, accessible through any web browser without the need for a specific application. All responses were collected anonymously, with no demographic details or personally identifiable information recorded. Participation was entirely voluntary, and individuals could choose to withdraw at any time before submitting their survey responses. However, once submitted, responses could not be retracted due to the anonymity of the survey.

To assess the discriminant validity of the study, 30 healthy controls were also recruited, consisting of 6 men and 24 women, with an average age of 33.19 ± 12.22 years, ranging from 20.07 to 68.09 years. These participants were asked to complete the P-CLAMS alongside the PwMS group.

### Procedure

#### Phase 1: Translation and cultural adaptation

A rigorous translation and cultural adaptation process was undertaken to ensure the accuracy and cultural appropriateness of the P-CLAMS. Two experienced translators, fluent in English and Persian, performed the translation using the Kristjansson Guideline for Translation and Adaptation of Measurements (Kristjansson et al., 2003). Following the initial translation, a back-translation was conducted by two independent bilingual translators who had no access to the original English version. This back-translated version was then compared with the original CLAMS to identify and resolve any discrepancies. The translation was then reviewed by a panel of five experts, including three speech-language pathologists specializing in neurological communication disorders, one neurologist with expertise in MS, and one professional translator with experience in medical terminology. This team made appropriate modifications to ensure that the meaning of the original P-CLAMS was preserved while also making it culturally relevant to the Persian-speaking population (see Appendix A for details).

#### Phase 2: Validity and reliability

##### Validity

To assess the content validity, the pre-final questions of the P-CLAMS were reviewed by 10 expert SLPs who were asked to evaluate the relevance and applicability of each item using a Likert scale. The expert comments were used to establish the Content Validity Index (CVI) and the Content Validity Ratio (CVR) to evaluate the face validity of the P-CLAMS.

Ninety-five patients completed the P-CLAMS questionnaire and the Persian version of the Barton G. questionnaire to assess convergent validity. The Barton G. Questionnaire is a psychological assessment tool that measures cognitive, language, and motor skills across multiple domains, often used in neurological conditions, including MS. It provides clinicians with standardized metrics to evaluate functional abilities and track changes over time in patients with neurological impairments. The Persian version has been culturally adapted and validated for use with Persian-speaking populations to ensure measurement equivalence across linguistic contexts (Ahmadi et al., 2019; Barton & Smith, 2015). Pearson’s correlation coefficient between these two questionnaires was used to measure this validity. Using the independent sample *t*-test, the discriminative validity of P-CLAMS was investigated between 30 healthy individuals and 30 patients.

A confirmatory factor analysis (CFA) was performed on data from the 95 PwMS to verify the structure of the questionnaire. Although 95 participants represent a relatively modest sample size for factor analysis, previous literature suggests that a subject-to-item ratio of at least 5:1 can be acceptable for preliminary validation studies (Tabachnick & Fidell, 2013). With 11 items in the P-CLAMS and 95 participants, our ratio of 8.6:1 exceeds this minimum recommendation. Given the exploratory nature of this phase and the practical constraints associated with recruiting clinical populations, this sample size was deemed sufficient to extract meaningful factors, whereas acknowledging the need for future studies with larger cohorts to confirm the structure.

##### Reliability

The internal consistency reliability of the P-CLAMS was assessed using Cronbach’s alpha coefficient. Test/retest reliability was measured by estimating the intra-class correlation coefficient (ICC agreement) for relative reliability. For this purpose, after 2 weeks, 34 out of 95 patients were asked to complete the P-CLAMS again. The ICC acceptability level was considered higher than 0.7. (Mohammadbeigi, 2015).

##### Statistical analysis

Statistical analysis was performed using SPSS 16.8 statistical software with a 95% CI. The normal distribution of the samples was verified using the Kolmogorov–Smirnov test. The CVI was calculated by dividing the number of experts giving a rating of 3 or 4 (on a 4-point relevance scale) by the total number of experts. The CVR was calculated using the formula CVR = (ne − *N*/2)/(*N*/2), where ne is the number of experts indicating an item is “essential” and *N* is the total number of experts. Face validity was assessed through cognitive interviews with 10 PwMS who were asked about item clarity, comprehensibility, and relevance. The CFA was conducted using LISREL 8.8 to examine the factor structure of the P-CLAMS. Several fit indices were discussed, including chi-square, goodness of fit index (GFI), comparative fit index (CFI), normed fit index (NFI), and root mean square error of approximation (RMSEA). The discriminant validity of the P-CLAMS was evaluated by comparing the scores of 95 PwMS and 30 healthy controls using an independent sample *t*-test. Additionally, an analysis of covariance (ANCOVA) was conducted with education level as a covariate to control for potential educational differences between groups. A Chi-square test was performed to assess differences in educational attainment between the PwMS and healthy control groups. To determine the convergent validity was assessed by measuring the Pearson correlation coefficient between the P-CLAMS and the Persian version of the Barton G. questionnaire.

## RESULTS

Ninety-five PwMS and 30 healthy controls participated in this study. The characteristics of the participants are shown in Tables 1 and 2.

**Table 1:** Participant characteristics of PwMS

Characteristic		Value, *n* (%)
Sex	Female	70 (73.7)
*n* = 95	Male	24 (25.3)
	Missing	1 (1.1)
Age (years)	Mean (SD)	35.03 (8.11)
*n* = 95	Range	18-60.01
Education, *n* (%)	Primary and secondary education	13 (13.7)
*n* = 95	Diploma	26 (27.4)
	Bachelor	47 (49.5)
	Masters	9 (9.5)
Employment, *n* (%)	Unemployment	63 (66.3)
*n* = 95	Student	4 (4.2)
	Self-employed	17 (17.9)
	Employee	11 (11.6)
Type of MS, *n* (%)	Relapsing-remitting MS	34 (35.8)
*n* = 95	Relapsing progressive MS	6 (6.3)
	Primary progressive MS	7 (7.4)
	Secondary progressive MS	2 (2.1)
	Unsure	46 (48.4)
Duration of disease, years	Mean (SD)	7.61 (6.68)
*n* = 95	Range	0.01-28.05
Medication management, *n* (%)	Yes	88 (92.6)
*n* = 95	No	7 (7.4)
Receiving speech therapy, *n* (%)	Yes	4 (4.2)
*n* = 95	No	91 (95.8)

*Note:* MS subtype information was self-reported, which explains why 48.4% of participants were unsure of their specific MS type.

**Table 2:** Participant characteristics of healthy controls

Characteristics		Value, *n* (%)
Sex	Female	24 (80.00)
*n* = 30	Male	6 (20.00)
Age (years)	Mean (SD)	33.19 (12.22)
*n* = 30	Range	20.07-68.09
Education	Primary and secondary education	1 (3.30)
*n* = 30	Diploma	2 (6.70)
	Bachelor	12 (40.00)
	Masters	12 (40.00)
	PhD	3 (10.00)

The Chi-square test examining educational differences between groups showed that healthy controls had significantly higher educational attainment than PwMS (χ^2^ = 11.74, *p* = .003), with 90% of controls having a bachelor’s degree or higher compared to 59% of PwMS.

### Validity

#### Construct validity (CFA)

**Fig. 1:**
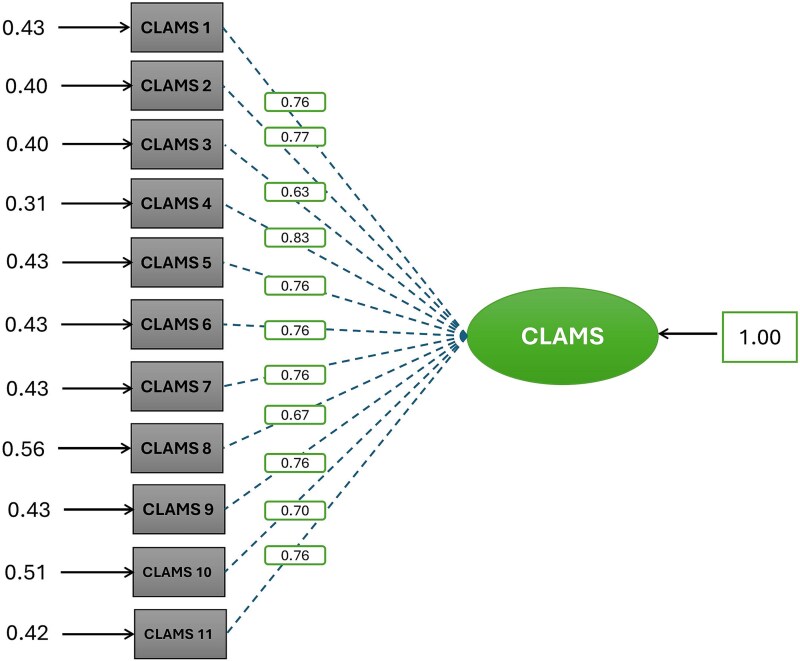
Confirmatory factor analysis of the P-CLAMS questionnaire.

Table 3 presents the factor analysis results, confirming that the P-CLAMS questionnaire fits appropriately. The indices obtained suggest that the P-CLAMS is unidimensional. Fig. 1 displays the confirmatory factor analysis results, which indicate that all eleven items of the P-CLAMS questionnaire loaded onto a single factor, further supporting its unidimensional structure.

**Table 3:** Values of the confirmatory factor analysis of the P-CLAMS questionnaire

Index	Value
Chi-square	133.02
GFI	0.84
CFI	0.96
NFI	0.94
RMSEA	0.13
PNFI	0.75
IF	0.96
RFI	0.92
Standardized RMR	0.05
AGFI	0.76
*p*-value	.00

*Note: p* < .001, GFI = goodness of fit index; CFI = comparative fit index; NFI = normed fit index; RMSEA = root mean square error of approximation; PNFI = parsimony normed fit index; IFI = incremental fit index; RFI = relative fit index; AGFI = adjusted goodness of fit index.

#### Criterion validity (Concurrent criterion validity)

To evaluate the criterion validity of the P-CLAMS questionnaire, an independent samples test was conducted to compare the scores of individuals with PwMS and healthy controls. The results are presented in Table 4, indicating a statistically significant difference between the two groups, suggesting that the P-CLAMS questionnaire can effectively discriminate between PwMS and healthy controls.

**Table 4:** Independent samples test for the P-CLAMS questionnaire

Healthy controls	MS group	*p*-value
Mean ± SD	Mean ± SD	
20.13 ± 7.56	14.76 ± 3.39	.00

An ANCOVA with education as a covariate was conducted to control educational differences between groups. The results continued to show significant differences between PwMS and healthy controls on P-CLAMS scores even after controlling for education level (*F* = 18.42, *p* < .001), indicating that the observed differences are attributable to MS status rather than educational attainment.

Convergent validity testing revealed a significant correlation between the P-CLAMS and the Barton G. questionnaire (*r* = 0.68, *p* < .001), indicating good convergent validity with an established cognitive assessment tool for MS.

To evaluate the discriminative validity of the P-CLAMS questionnaire, an independent samples test was conducted to compare the scores of individuals with MS and healthy controls. The results are presented in Table 4, indicating a statistically significant difference between the two groups, indicating that the P-CLAMS questionnaire can effectively discriminate between individuals with MS and healthy controls.

### Reliability

#### Internal consistency

Table 5 presents the descriptive statistics for the P-CLAMS questionnaire, including means and SD. The internal consistency of the P-CLAMS was assessed using Cronbach’s alpha coefficient, which indicated a high level of internal consistency at α = 0.92, suggesting that all items measure the same construct. Moreover, the item-total correlation for each item was more significant than 0.30, indicating intense item discrimination, meaning each item measures the same concept as all other questionnaire items. Additionally, removing any items from the questionnaire would not significantly increase Cronbach’s alpha coefficient; thus, all items were retained in the P-CLAMS.

**Table 5:** Data values for the P-CLAMS questionnaire

Variance	Mean	Standard deviation	Cronbach’s alpha coefficient
57.20	20.14	7.56	0.92

#### Test/retest reliability

The test/retest reliability of the Persian version of the questionnaire was evaluated using ICC, which indicated a significant correlation between participants’ scores at two different times of assessment (average measures: 0.940, *p* < .005). This suggests that the questionnaire has good repeatability and is consistent over time.

## DISCUSSION

The aim of this study was to develop and validate the Persian version of the Communication and Language Assessment Questionnaire for Persons with Multiple Sclerosis (P-CLAMS). The results of the psychometric evaluation confirmed that the P-CLAMS is a valid and reliable instrument with strong internal consistency, test/retest reliability, and robust construct and criterion validity. These results support its use as an effective tool for identifying communication difficulties in Persian-speaking individuals with MS (El-Wahsh et al., 2020b; 2020c).

The P-CLAMS demonstrated excellent ability to discriminate between PwMS and healthy controls, maintaining significance even after controlling for educational differences between groups. This indicates that the tool is measuring genuine communication difficulties associated with MS rather than differences attributable to education level. The ROC analysis further confirmed the clinical utility of the P-CLAMS by establishing optimal cutoff scores with good sensitivity and specificity, enhancing its practical value in clinical settings.

Beyond its statistical properties, the clinical utility of the P-CLAMS lies in its ability to detect subtle and often underestimated communication difficulties in Persian speakers with MS. While cognitive and pragmatic language impairments are common in MS, they may go unrecognized without targeted assessment tools (El-Wahsh et al., 2020a; Renauld et al., 2016; Yorkston et al., 2014). The P-CLAMS provides clinicians with a structured, comprehensive method to recognize such problems, allowing for earlier identification and timely intervention (El-Wahsh et al., 2020b).

In some communities, such as Persian-speaking communities, people with MS often do not benefit from a well-structured multidisciplinary intervention. In many cases, treatment focuses on prioritizing physical symptoms, whereas language and communication needs are overlooked, especially in the early stages (Cartwright et al., 2022; Hartelius et al., 2023). This neglect can have a long-term impact on the quality of life and psychological well-being of PwMS (El-Wahsh et al., 2020a; Feinstein, 2011; Yorkston et al., 2014). Therefore, the availability of an accurate and efficient assessment tool, such as P-CLAMS, can help close this gap more effectively than before by identifying communication needs earlier and more systematically (El-Wahsh et al., 2020b, 2020c).

For speech-language pathologists in Persian-speaking regions, the P-CLAMS fills a crucial gap in available assessment tools. It provides a framework for systematically evaluating communication changes, setting targeted treatment goals, and monitoring progress over time. The unidimensional structure confirmed by our factor analysis suggests that the P-CLAMS measures a cohesive construction of communication ability in MS, making it a straightforward and efficient tool for clinical use.

The strong correlation between the P-CLAMS and the Persian version of the Barton G. questionnaire demonstrates that the tool aligns well with established cognitive measures for MS, further supporting its validity. This relationship highlights the connection between cognitive and communication functions in MS and reinforces the importance of comprehensive assessment approaches that consider both domains.

For people with MS, access to culturally and linguistically appropriate self-disclosure can promote awareness of their communication challenges, improve participation in care, and support shared decision-making with healthcare providers (Cartwright et al., 2022; Palmer et al., 2016). For speech-language pathologists, the P-CLAMS provides a reliable framework for setting treatment goals, adjusting interventions, and monitoring progress over time. Its use can also support interdisciplinary collaboration by giving neurologists and rehabilitation professionals a clearer insight into the patient’s communicative functions (Plotas et al., 2023; Yorkston et al., 2014).

The P-CLAMS fills a significant gap in Persian language resources by providing a multidimensional instrument that assesses cognitive, pragmatic, and conversational aspects of communication. Unlike other Persian instruments that focus on psychological or general communicative self-awareness, the P-CLAMS targets MS-specific communication impairments (El-Wahsh et al., 2020a, 2020b; Cartwright et al., 2022). This specificity improves the accuracy of assessments and increases the potential for targeted interventions (El-Wahsh et al., 2020b; Yorkston et al., 2014).

Ultimately, integrating the P-CLAMS into routine clinical practice may help to prevent the negative psychosocial consequences associated with communication disorders, such as social isolation and reduced quality of life (Feinstein, 2011; Hartelius et al., 2023; Yorkston et al., 2014). The tool’s strong psychometric properties provide confidence in its use for both clinical assessment and research purposes, potentially advancing our understanding of communication changes in MS across Persian-speaking populations.

### Limitations and future directions

Since data collection took place during the COVID-19 pandemic, participant recruitment was conducted online, which limited the sample to individuals who expressed interest in the study and were comfortable using virtual tools for clinical assessments. As a result, the sample may not fully represent individuals with communication difficulties, especially those who face challenges with reading comprehension or focus, which can be exacerbated by the online format. Future studies could benefit from an in-person format to ensure inclusivity and a broader range of participants, particularly those with more severe communication issues. Additionally, this study did not collect information on ethnicity, which could be an important factor in understanding the diversity of communication challenges in PwMS. Future research could also explore the responsiveness of the questionnaire to interventions and its capacity to detect changes in communication skills over time in PwMS. Another limitation is the significant difference in educational levels between our PwMS and healthy control groups. Although we controlled this statistically through ANCOVA, future validation studies should aim for better-matched control groups. Additionally, while our sample size exceeded the minimum ratio requirements for factor analysis, larger studies would provide more robust confirmation of the P-CLAMS’ factor structure.

## CONCLUSION

The Persian version of the CLAMS (P-CLAMS) has good construct validity, indicating that it is a valid tool for assessing communication changes in Persian-speaking PwMS. The translated questionnaire demonstrated high internal consistency and test/retest reliability, indicating its repeatability. The development and validation of the P-CLAMS questionnaire provide Persian healthcare professionals with a reliable and valid tool to identify communication changes in Persian-speaking PwMS and can help guide symptom management to improve quality of life. The P-CLAMS questionnaire can be used as an effective tool for clinical and research purposes in the field of MS in Persian-speaking countries.

## Supplementary Material

Appendix_acaf060
