# Supplementary material for: Translation and Validation of the Persian Version of the Communication and Language Assessment Questionnaire for Persons with Multiple Sclerosis (P-CLAMS)
Source: Arch Clin Neuropsychol. 2025 Jun 30;40(8):1614–20. doi: 10.1093/arclin/acaf060 (PMC12644053; doi:10.1093/arclin/acaf060)
Supplement: Appendix_acaf060 [file appendix_acaf060.docx]

Appendix A

**Original version of CLAM**

**Communication and Language Assessment questionnaire for people with Multiple Sclerosis (CLAMS)**

The following questions ask about specific aspects of your communication and language. For each question, please select the response option that best applies to you. Make sure you consider ALL communication situations in your daily life (e.g., family, social, and work situations).

During the past 4 weeks, when talking to others, how often did you:

|  | Never or rarely (1) | Sometimes (2) | Often (3) | Usually or always (4) |
| --- | --- | --- | --- | --- |
| 1. Have difficulty thinking of the particular word you want? |  |  |  |  |
| 1. Have difficulty remembering your train of thought as you are speaking? |  |  |  |  |
| 1. Use a lot of vague or empty words, such as “you know what I mean,” instead of the right word? |  |  |  |  |
| 1. Find it difficult to remember information on the tip of your tongue? |  |  |  |  |
| 1. Find it difficult to convey precisely what you mean? |  |  |  |  |
| 1. Leave out important details? |  |  |  |  |
| 1. Find it difficult to concentrate enough to understand what is being said? |  |  |  |  |
| 1. Find it difficult to remember recent conversations? |  |  |  |  |
| 1. Find it difficult to keep track of the main details of conversations? |  |  |  |  |
| 1. Find it difficult to put ideas together in a logical way? |  |  |  |  |
| 1. Hesitate, pause, or repeat yourself? |  |  |  |  |

Persian Version CLAMS

(P-CLAMS)

| **معمولا یا همیشه (4)** | **اغلب (3)** | **گاهی اوقات (2)** | **به ندرت یا هرگز (1)** |  |
| --- | --- | --- | --- | --- |
|  |  |  |  | 1.آیا هنگام فکر کردن در مورد کلمه ی خاص مشکل دارید؟ |
|  |  |  |  | 2. آیا هنگام صحبت کردن، در به یادآوری توالی افکار خود مشکل دارید؟ |
|  |  |  |  | 3. آیا به جای واژه های صحیح، از واژه های مبهم مانند" می دانید منظورم چیست؟" استفاده می کنید؟ |
|  |  |  |  | 4. آیا در به یاد آوردن اطلاعاتی که نوک زبان شما هستند، با مشکل رو به رو هستید؟ |
|  |  |  |  | 5.آیا انتقال دقیق منظورتان برای شما دشوار است؟ |
|  |  |  |  | 6. آیا جزئیات مهم را فراموش می کنید؟ |
|  |  |  |  | 7.آیا تمرکز کافی برای درک آنچه گفته می شود، برای شما دشوار است؟ |
|  |  |  |  | 8.آیا به یاد آوردن گفت و گو های اخیر برای شما دشوار است؟ |
|  |  |  |  | 9.آیا پیگیری جزئیات اصلی مکالمات برای شما دشوار است؟ |
|  |  |  |  | 10.آیا یکپارچه کردن ایده ها به روش منطقی برای شما دشوار است؟ |
|  |  |  |  | 11. آیا هنگام صحبت، من من یا مکث می کنید؟ و یا گفته ی خود را تکرار می کنید؟ |

|  | Never or rarely (1) | Sometimes (2) | Often (3) | Usually or always (4) |
| --- | --- | --- | --- | --- |
| 1. Do you have difficulty thinking of particular words you want? |  |  |  |  |
| 1. Do you have difficulty remembering the train of thought as you are speaking? |  |  |  |  |
| 1. Do you use a lot of vague or empty words, such as “you know what I mean,” instead of the right word? |  |  |  |  |
| 1. Do you Find it difficult to remember information on the tip of your tongue? |  |  |  |  |
| 1. Is it difficult for you to convey precisely what you mean? |  |  |  |  |
| 1. Do you leave out important details? |  |  |  |  |
| 1. Is it difficult for you to concentrate enough to understand what is being said? |  |  |  |  |
| 1. Is it difficult for you to remember recent conversations? |  |  |  |  |
| 1. Is it difficult for you to keep track of the main details of conversations? |  |  |  |  |
| 1. Is it difficult for you to put ideas together in a logical way? |  |  |  |  |
| 1. Do you hesitate, pause, or repeat yourself? |  |  |  |  |

**Version of the P-CLAMS translated into the English system**
